# Methods of Non-Invasive In Vivo Optical Diagnostics in the Assessment of Structural Changes in the Skin Induced by Ultraviolet Exposure in an Experimental Model

**DOI:** 10.3390/diagnostics11081464

**Published:** 2021-08-12

**Authors:** Dmitry Kulikov, Mikhail Makmatov-Rys, Irina Raznitsyna, Polina Glazkova, Anastasiia Gerzhik, Alexey Glazkov, Viktoriya Andreeva, Darya Kassina, Dmitry Rogatkin

**Affiliations:** 1 Department of Experimental and Clinical Research, Moscow Regional Research and Clinical Institute (“MONIKI”), 129110 Moscow, Russia; zdolsk2@gmail.com; 2 Medical Faculty, Moscow Region State University, 141014 Mytishi, Russia; 3 Laboratory of Medical and Physics Research, Moscow Regional Research and Clinical Institute (“MONIKI”), 129110 Moscow, Russia; raznitsynaia@yandex.ru (I.R.); polinkul@mail.ru (P.G.); gerjik18@yandex.ru (A.G.); staaglz@gmail.com (A.G.); vertebra1496@mail.ru (D.K.); rogatkin@medphyslab.com (D.R.); 4 Department of Oral and Maxillofacial Surgery, Moscow Regional Research and Clinical Institute (“MONIKI”), 129110 Moscow, Russia; viktoriaa@yandex.ru

**Keywords:** ultraviolet radiation, skin, in vivo, photoaging, erythema, noninvasive diagnostics, autofluorescence

## Abstract

Background: This paper demonstrates the use of optical diagnostic methods to assess the dynamic skin changes observed in acute and chronic exposure to ultraviolet (UV) radiation in vivo. Methods: Firstly, in order to initiate photoaging (chronic UV exposure), animals (*n* = 40) were divided into two groups: chronic UV exposure (*n* = 30), and control (*n* = 10; without irradiation). Photoaging in animals was induced by chronic repeated exposure to UVA radiation three times per week, for 12 weeks continuously, while the UV dose increased stepwise over the course of the experiment (55 minimal erythema doses (MED) in total). Laser fluorescence spectroscopy (LFS), optical tissue oximetry (OTO), laser Doppler flowmetry (LDF), and optical coherence tomography (OCT) of the shaved dorsum skin were performed regularly, once per week until the conclusion of the study. At 0, 5, and 12 weeks of the experiment, histological examination of animal tissues using hematoxylin/eosin and Masson’s trichrome staining was performed. At the second stage, erythema was induced in mice (*n* = 15) by acute UV exposure at high doses. The colorimetric assay of the image from a digital RGB camera was used to evaluate the erythema index. Results: The tissue content index *η_collagen_* of collagen was appropriate for the characterization of skin photoaging. Significant differences (*p* < 0.05) in *η_collagen_* were found between the control and photoaging groups from the 5th to the 9th week of the experiment. In addition, the rate of collagen degradation in the control group was about half that of the photoaging group. This marker allows the differentiation of photo- and chronoaging. OCT revealed the main optical layers of the skin in compliance with the histological pattern. The analysis of the RGB camera images provided visualization of the acute skin reaction to UV radiation. Conclusions: This study demonstrates the applicability of optical methods for the quantitative assessment of acute and chronic skin effects of UV exposure in vivo.

## 1. Introduction

Ultraviolet (UV) radiation is a component of the total spectrum of solar radiation, and results in combined complex effects on living organisms and tissues. The UV radiation range is usually divided into UVC (100–280 nm), UVB (280–320 nm), and UVA (320–400 nm). Each of these ranges possesses varying degrees of penetrating power into the skin. While UVB and UVC photons are substantively absorbed in the epidermis, UVA rays can penetrate into the dermis. In a short time interval, UV radiation contributes to the formation of vitamin D in the skin, has a bactericidal effect (primarily UVC), and at high doses can lead to the formation of UV-induced erythema (“sunburn”). On the other hand, during prolonged exposure, UV induces the formation of malignant tumors, degenerative skin changes (photoaging), and immunosuppression [1].

The processes of interaction of ultraviolet photons of various energies have been described in detail in a number of publications [2,3]. The fundamental damaging effect of UV radiation of any range is a result of direct or indirect (due to the generation of reactive oxygen intermediates) DNA damage, which can be followed by alterations to DNA-chain repair, cell cycle control, and apoptosis [4]. The accumulation of genetic changes in combination with UV-induced immunosuppression can trigger the development of basal-cell/squamous-cell skin cancer or melanoma [5]. For example, UV-induced mutations in the p53 tumor-suppressor gene have been described in squamous-cell carcinoma [6,7]. Moreover, exome analysis has revealed strong genetic evidence for the direct mutagenic role of UV radiation in the pathogenesis of melanoma [8]. UV also causes protein degradation, the formation of more complicated biological molecules with new physical and chemical properties, and reactive intermediates. In particular, UV radiation induces the release of histamines by mast cells and acetylcholine by keratinocytes, and these mediators, upon entry into the bloodstream, affect vascular, muscular, and nerve fiber tone [9]

In 1907, the first data on acute skin reactions to UV radiation, and clinical cases with successful UV-treatment of skin diseases, began to appear. The therapeutic effect of UV was attributed mainly to vasodilation and increase in local blood perfusion [10,11]. Over more than a century of researching the influence of UV light on the skin, other properties of phototherapy—namely, anti-inflammatory, immunosuppressive, antiproliferative, and antifibrotic effects—have been discovered [12]. Thus, taking into account such an ambivalent effect of UV light on the skin, modern medicine faces the task of personalized adjustment of therapeutic radiation doses to minimize possible adverse effects.

The study of skin reactions to both acute and chronic UV exposure is of scientific and practical interest. An acute skin reaction to UV is exhibited in the formation of erythema. In dermatology, skin sensitivity to UV is assessed by the index of the minimal erythema dose (MED)—the lowest dose of UV radiation that causes the development of a minimal erythema [13]. Extended and repeated UV exposure is an important factor affecting the severity and rate of age-related skin degeneration. Photoaging manifests itself in the form of dryness and thinning, slowing down the processes of skin regeneration, and is associated with the degradation of collagen, elastin, and hyaluronic acid in the skin [3]. In clinical practice, the intensity of photoaging and acute UV damage (UV erythema) is typically determined visually and indirectly, which is subjective due to the high variability of the obtained data, misdiagnoses, and non-accurate data collection for clinical trials.

The prospects of using non-invasive optical diagnostics to assess the effects of acute and chronic UV-damage to the skin are actively discussed in scientific literature. Visible and near-infrared radiation, as well as high-frequency ultrasonic radiation [14], allow the quick and noninvasive performance of skin introscopy with high spatial resolution. At the same time, the degree of photoaging is assessed by structural alterations in the skin—in particular, by changes in the density and structures of collagen fibers, keratinocytes, and the thickness of the epidermis [15,16]. However, the analysis of such data is subjective, depending on the experience and qualifications of the researcher.

It is possible to assess objectively and quantitatively the parameters of local skin blood flow, which reflects the persistent vasodilation of the dermal vessels induced by prolonged UV exposure, using laser Doppler flowmetry and optical tissue oximetry. Laser fluorescence spectroscopy allows us to assess changes in the biochemical composition of tissues. Most often, as part of the study of photoaging processes, authors examine the changes in the fluorescence spectra of collagen and elastin [17,18]; less often, those of tryptophan [19]. Using colorimetric assays, it is possible to quantify the MED based on the results of test irradiations, followed by erythema index evaluation [20].

Animal models represent an optimal choice to study the effects of UV exposure on the skin. The literature describes in detail a number of models of UV-induced erythema and photoaging; these have been mentioned since the 1980s [21,22]. In order to induce photoaging, repeated irradiation of animals should be conducted for an average of 10–12 weeks. The main advantages of the in vivo approach include the possibility of eliminating the influence of UV-induced skin changes due to side factors (the level of pigmentation, influence of skin diseases, diet, ambient light, etc.), accurate selection of the dose and geometry of UV radiation, as well as the possibility of conducting a verifying histological examination at any stage of the experiment.

Today, a large number of publications devoted to the objective assessment of UV-induced skin changes using noninvasive optical methods provides mostly descriptive and non-quantitative data, which do not allow us to create diagnostic algorithms and further translate them into clinical practice. Moreover, there is a lack of histological verification of optical data in the published studies. Thus, a quantitative analysis of both morphological and functional changes to the skin skin using modern optical methods in conjunction with reference histological examination remains relevant. Therefore, the aim of this work was to investigate possible objective markers for the in vivo assessment of acute and chronic UV-induced skin changes using optical noninvasive methods.

## 2. Materials and Methods

### 2.1. Modeling and Assessment of Photoaging In Vivo Using Optical Methods

It is known that the main damaging factor leading to photoaging is extended and repeated exposure to sunlight, mostly including UVA radiation [23].

#### 2.1.1. Laboratory Animals and Experimental Design

Within the framework of the project, an experimental study was carried out on white ICR mice—males, aged 6 weeks, weighing 25–38 g (*n* = 35), receiving a balanced granular feed containing no fluorophores. The animals had free access to drinking water. Mice were kept at a temperature of 21–23 °C and humidity of 50–65%, under a 12 h dark/light cycle. The duration of the mouse quarantine period was 10 days.

The animals (*n* = 35) were divided into 2 groups: the 1st group for photoaging (*n* = 25), and the 2nd group for chronoaging (*n* = 10; control). This ratio of test group sizes is due to the better survival of mice in the control group.

The experiment was based on a modified version of the in vivo skin photoaging model [24]. This model is used not only to model chronic UV-induced skin damage, but also to study photocarcinogenesis, as well as to assess the effectiveness of photoprotective and antioxidant substances [25]. Photoaging in animals was initiated by chronic repeated exposure to UVA radiation, since over 95% of solar UV radiation on the Earth’s surface is composed from this waveband. Thirty animals were exposed to UVA three times a week for 12 weeks. LDF, OTO, and OCT of the dorsum skin in vivo were performed once a week until the experiment was finished.

**Figure A1:**
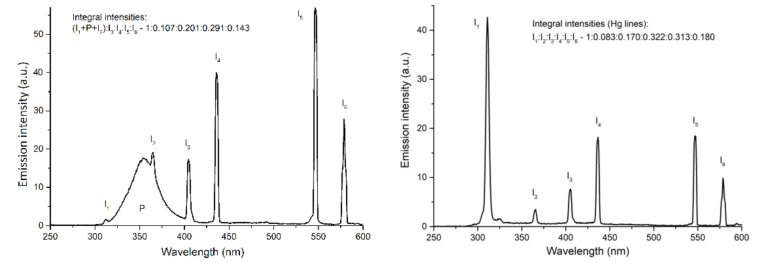
UVA (**left**) and UVB (**right**) radiation spectra of used sources.

The hair covering the dorsal zone was carefully removed (Veet depilatory cream) 24 h prior to the irradiation sessions and optical diagnostics [26]. The described method of chemical hair removal in some cases could cause mild pale erythema, which usually regressed 6–12 h after the procedure. The irradiation was performed using a therapeutic UVA source—YUTEC-solarium gk-480-s8/515. The source’s radiation spectrum was detected using an S100 UV spectrometer (SOLAR LS, Minsk, Belarus), and is shown in Figure A1. The intensity of UV radiation at the distance between the source and the skin of the dorsum (about 30 cm) was measured weekly, controlled using a TKA-PKM spectroradiometer (UV-meter) (LLC “NTP” “TKA”, St. Petersburg, Russia), and was found to be 4.5 mW/cm^2^. During UV irradiation, a cage with non-anesthetized free-moving animals (*n* = 10 per session) was placed directly under the UV radiation source. In a preliminary experiment using this source, a minimal erythema dose (MED) of 300 mJ/cm^2^ was established for ICR mice. During the experiment, the dose of UV radiation increased stepwise: 1/3 MED per day for week 1 (cumulatively 1 MED per week), 2/3 MED per day for week 2 (cumulatively 2 MED per week), 1 MED + 4/3 MED + 5/3 MED (cumulatively 4 MED per week) for week 3, and then 2 MED per day 3 times a week (cumulatively 6 MED per week) for weeks 4–12. The animals were exposed to increasing doses of UV instead of a constant dose in order to induce evident skin photoaging in the planned experimental period. The algorithm of UV dose increase in the framework of the photoaging mice model is well established, and is described in previous publications [17,21,22]. Irradiation was performed 3 times per week—on Mondays, Wednesdays, and Fridays. The total UV dose for the entire period of the experiment was 55 MED. To assess morphological changes in the processes of photo- and chronoaging, 3 animals were euthanized from the experimental group along with 1 mouse from the control group, and a dorsum skin biopsy was performed after 5, 10 and, 12 weeks of the experiment. The age-matched control group (*n* = 10) was not exposed to UV radiation, and the chronoaging processes were assessed in this group. All measurements, the withdrawal of animals from the experiment, and the collection of histological samples in the control group were carried out according to the above-mentioned algorithm.

#### 2.1.2. Optical Diagnostics

**Figure A2:**
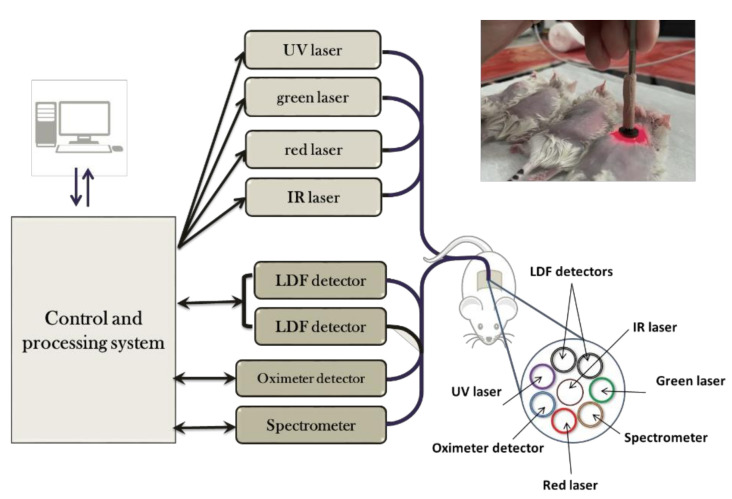
LAKK-M scheme and the position of the fiber-optic probe tip when measuring fluorescence spectra.

Endogenous fluorescence of animal skin collagen was studied via LFS in vivo using the LAKK-M diagnostic complex (SPE “LAZMA”, Moscow, Russia). In the device, low-power radiation from a laser source with a wavelength of *λ_e_* = 365 nm was delivered to the surface of the living tissue through the illumination fiber of the fiber-optic probe. The secondary radiation (backscattered and fluorescent) that fell into the receiving fiber of the probe was recorded with a spectrometer, while the backscattered part of the radiation was reduced by a light filter by about 1000 times. The diameters of the illumination and receiving fibers were 100 microns; the distance between the fibers was about 2 mm. The LAKK-M scheme is shown in Figure A2. The fluorescence spectra of collagen—the main structural element of the skin—and porphyrins—which can be taken as markers of inflammation [27]—were analyzed.

Quantitative assay of the fluorescence was carried out using the values of tissue content indices *η_fluorophore_*, calculated by Equation (1):

(1) $$\eta_{fluorophore}=\frac{I_{f}}{I_{f}+I_{bs}},$$

where *I_f_* is the fluorescence intensity at the wavelength of the maximum fluorescence of the fluorophore, and *I_bs_* is the intensity of backscattered radiation.

**Figure A3:**
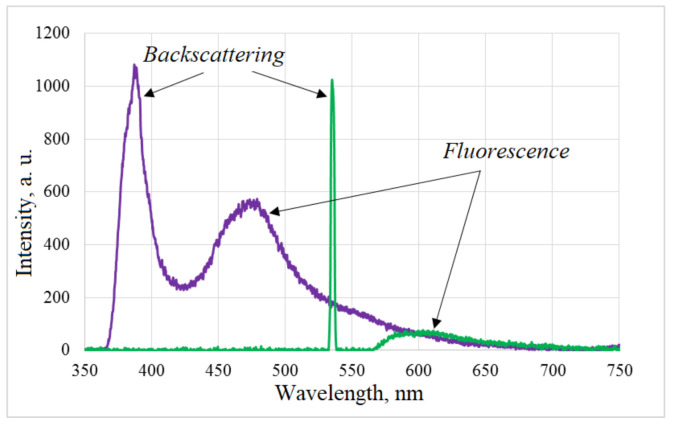
Examples of registered fluorescence spectra at *λ_e_* = 365 nm and *λ_e_* = 535 nm.

Porphyrin fluorescence is clearly discernible in the red waveband. The fluorescence spectrum has two characteristic peaks at the wavelengths of 630 and 710 nm [28]. In the current work, the fluorescence intensity was recorded at the wavelength of *λ_f_* = 630 nm when excited by a laser source with a wavelength of *λ_e_* = 535 nm. Collagen fluorescence was recorded at the wavelength of *λ_f_* = 450 nm. This choice was due to the use of a diode as an exciting source with a central wavelength of 365 nm, and the spectrum that partially overlaps the maximum of collagen fluorescence—420 nm. According to [29], the fluorescence of collagen can also be detected in the longer wavelength part of the spectrum. The *I_bs_* value is evaluated to maximum in the region of 380 nm, since the optical filter almost completely cuts off the shorter wavelength part of the spectrum (Figure A3).

Weekly measurements of endogenous fluorescence for each animal were carried out at five points in the skin area under examination, and then the indices were averaged.

The blood flow parameters were assessed at the tissue level using the LAKK-M device. In the “microcirculation” mode, the device assesses laser Doppler flowmetry (LDF), and continuously registers the baseline perfusion (BP), which is measured in perfusion units (p.u.). LDF measures reflected and scattered laser light. The resulting perfusion signal is proportional to the velocity and concentration of moving red blood cells.

In parallel, the relative volume of all fractions of hemoglobin (total hemoglobin) in a tested tissue’s volume (*V_b_*) was recorded using optical tissue oximetry [30]. Indices from five points of the skin area under examination were recorded for 15–20 s, then averaged over the registration time.

Optical coherence tomography (OCT) is a method of noninvasive imaging of the microstructure of biological tissues. OCT is based on the registration of light reflected from the structures of the object, followed by the analysis of the interference of recording and reference beams. This method is widely used in scientific research to visualize skin structures, both in healthy conditions and in dermatological diseases [31].

At the measurement timepoints of the experiment, all animals were subjected to OCT of the skin area under examination at one point, in the center of the dorsum, using an OCT1300U device (BioMedTech, Moscow, Russia). The results of the OCT study are presented as images of skin structures. The depth of the studied volume was about 700 microns. Analyzing the OCT image, the integrity of the structural layers, their relative position, and their thickness are assessed. OCT images of mouse skin were interpreted by comparing them with histological cross-section images. The specific optical layers that are usually segregated in the human skin were taken into account [32].

#### 2.1.3. Histological Examination

Histological examination is a reference method for verifying the pathological condition of the skin, which is why the parameters of the optical diagnosis of the skin were compared with the obtained morphological data.

At 0, 5, and 12 weeks of the experiment on the animals, histological examination of tissues was performed. The animals were euthanized with a lethal intraperitoneal dose of 5 mg/kg tiletamine hydrochloride/zolazepam hydrochloride (Zoletil 100, Virbac Sante Animale, Carros, France) and 5 mg/kg xylazine hydrochloride (Rometar, Bioveta, a. s., Ivanovice na Hané, Czech Republic), and a skin biopsy specimen of 1 × 1 cm^2^ was trimmed out and fixed in 10% formalin solution. The obtained samples were dehydrated in ascending concentrations of alcohols, the material was embedded into Histomix^®^, and the seven-micron-thick sections of the specimen were prepared. Histological transaction was carried out according to the standard protocol, followed by staining of the sections with hematoxylin/eosin. During histological examination of hematoxylin/eosin-stained preparations, the following parameters were assessed: the condition of the epidermis, the presence of “sunburn” cells (keratinocytes in the process of UV-induced apoptosis), inflammatory changes in the dermis and in subcutaneous adipose tissue, the thickness of the epidermis and dermis, the distribution of mast cells, and the structure of collagen and elastin fibers.

Masson’s trichrome staining was used for further quantitative assessment of collagen fibers in the skin subjected to photo- and chronoaging. To assess the severity of collagen accumulation, the sections were deparaffinized in xylene and rehydrated in ethanol, followed by fixation in Bouin’s fixative for 1 h at 55 °C. The nuclei were stained with a solution of iron chloride, and then the collagen was stained with an alcoholic solution of hematoxylin and a solution of trichrome. The total collagen content was calculated as a percentage of the aniline blue staining of the tissue divided by the total biopsy specimen area, using color threshold filtering and the MATLAB platform. The assessment of the specimens was carried out via light microscopy at 100-, 400-, and 500-fold magnification (ZEISS Axiolab A1 microscope, Zeiss Microscopy, Jena, Germany).

#### 2.1.4. Statistical Analysis

The normality of the quantitative variables’ distribution was checked using the Shapiro–Wilk test. Arithmetic means and standard deviations were calculated as parameters of descriptive statistics. Due to the limited number of observations, the quantitative variables in the two groups were compared using the Mann–Whitney test. The dynamics of the fluorescence parameters were compared by linear regression modeling and analysis of the coefficients of linear functions with 95% two-sided confidence intervals. Statistical analysis was performed using the IBM SPSS Statistics v25 (IBM Corp., Endicott, NY, USA) and Microsoft Excel 2019 (Microsoft Corp., Albuquerque, NM, USA) software. The differences were considered statistically significant when the *p*-value was less than 0.05 (*p* < 0.05).

### 2.2. In Vivo UV Erythema Modeling and Colorimetric Assay

As noted above, erythema is an acute reaction to UV over-irradiation, accompanied by a complex of vascular changes.

#### 2.2.1. Laboratory Animals and Experimental Design

Skin erythema modeling was carried out by a single irradiation of the pre-depilated half of the dorsal skin of the laboratory animals. The study involved ICR mice (N = 15). Laboratory animals were divided into three groups: the 5 mice of the first group received a UVB dose of 9450 mJ/cm^2^, the 5 mice of the second group received a dose of 13,500 mJ/cm^2^, and the 5 mice of the third group received a dose of 18,450 mJ/cm^2^. The irradiation was performed using a Dermalight 500-1 series therapeutic UV source (Dr. Hönle Medizintechnik GmbH, Gilching, Germany) with a wavelength of 311 nm (Figure A1). The intensity of UV radiation at the distance between the source and the skin of the dorsum of the animals was 150 W/m^2^. Such high radiation doses were necessary for clinically apparent UV-induced erythema modeling, and are due to the poor erythema-promoting effect of narrow-band (NB) UVB with a wavelength of 311 nm; it is reported that the MED for NB-UVB in different mouse strains is significantly higher than for broad-band (BB) UV, and can reach 1350 mJ/cm^2^ [33,34]. In our preliminary experiments, the NB-UVB MED for ICR mice was 700 mJ/cm^2^.

Prior to the irradiation session, the mice’s dorsal skin was depilated, but the right side of the dorsum remained intact and was protected from irradiation. During irradiation, the animals were anesthetized with an intraperitoneal injection of 50 μg/kg tiletamine hydrochloride/zolazepam hydrochloride (Zoletil 100, Virbac Sante Animale, France) and 50 μg/kg xylazine hydrochloride (Rometar, Bioveta, a. s., Ivanovice na Hané, Czech Republic). The photos were taken before exposure, 24 and 48 h after irradiation. During the photography sessions, animals were not anesthetized, in order to improve their survival. Therefore, mice on different images are in slightly different positions, but the distance from the camera to the subject, camera parameters, and light source power were fixed.

#### 2.2.2. Evaluation of the Erythema Index

To assess changes in the irradiated skin area, the erythema index was evaluated using digital color imaging and photography based on colorimetric assay of the image from a digital RGB camera [35,36,37]. UV irradiation, implemented in the study, leads to the development of erythema in animal skin. Therefore, the erythema index evaluated from the photographs in real-time mode could serve as an indicator of structural and functional changes in the skin.

**Figure A4:**
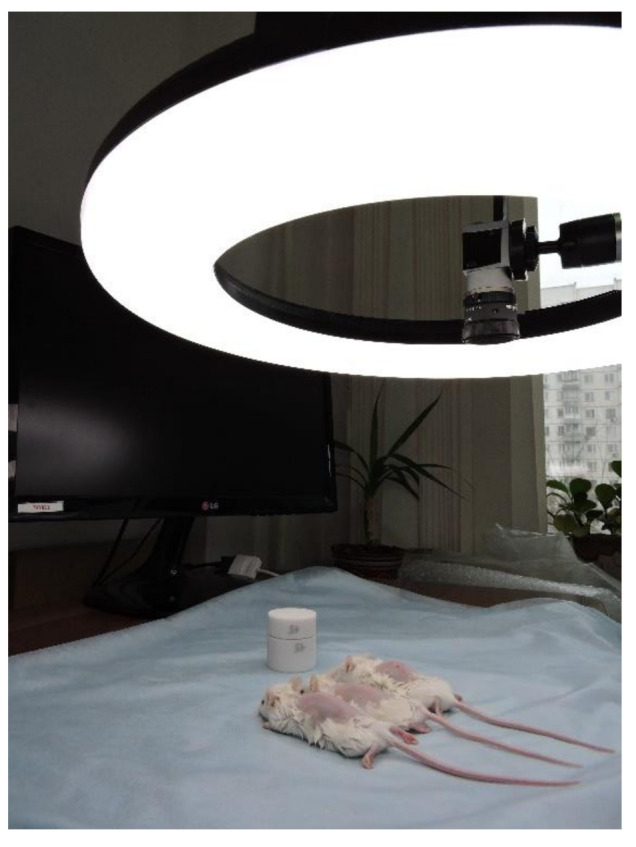
Position of the RGB camera and the light source during colorimetric assay of erythema.

Photos of animals’ skin were taken using a Basler acA1300-200uc USB 3.0 camera with the ON Semiconductor PYTHON 1300 CMOS sensor, at a resolution of 1028 × 1024. When the image processing (gamma correction and auto balance adjustment) was disabled, the images were saved in raw format, with an encoding depth of 8 bits. In order to manually adjust the white balance during shooting, a white phantom evenly scattering visible light was placed in the frame. The light source was an Aura Helle FC-480 SE Multicolor (RGB) Max ring lamp with a maximum luminous flux of 9600 lm. All measurements were carried out at 20% of the maximum power. The color temperature of the broadband radiation source was 4500 K. The distance between the radiation source and the mouse was 20 cm. The camera was mounted on a tripod and installed at a right angle to the shooting plane (Figure A4). The exposure time was 5 ms, and the aperture opening ratio was f1.4.

To evaluate the erythema index, a conversion to the L*a*b* color space was applied. The a* coordinate was taken as the value of the erythema index, since it reflects the degree of redness of the skin [35]. The conversion equations from RGB to L*a*b* are described in the literature [38]. The relative erythema index ∆a*, expressed as a percentage, was calculated using the equation:

(2) $$∆a^{*}=\frac{a_{AS}^{*}-a_{NS}^{*}}{a_{NS}^{*}}\times 100,$$

where a*_AS_ is the erythema index of the biopsy skin specimen under study, and a*_NS_ is the erythema index of intact skin. By way of image processing, the nonirradiated right region of the mouse’s dorsum was taken as an area of intact skin.

To determine a*_AS_ and a*_NS_, images of mice were converted from RGB to the L*a*b*color space. Areas of 40 × 40 pixels in the intact and irradiated regions were selected within the frames of the images. The mean values of the color space coordinate a* inside the selected areas were taken for a*_AS_ and a*_NS_.

To assess the ∆a* index dynamics after irradiation, a statistical analysis with pairwise comparisons according to Tukey’s criterion was carried out. For this purpose, the average values of the ∆a* index (the average over 4 ∆a*values calculated from one photo of one mouse from 4 different 40 × 40 pixels areas of the dorsal skin) for each of the study days were determined for each of the 5 mice in the groups. Thus, to evaluate the statistical differences in the index ∆a*, 15 ∆a* values (5 for each day of the study) were sampled within each of the 3 groups. The differences were considered significant at the *p*-value level < 0.05. Similarly, the evaluation of the differences in the a* index between groups that received different radiation doses was performed. 15 ∆a* values (5 per group) were sampled for each of the 3 days of the study.

## 3. Results and Discussion

### 3.1. Photoaging

#### 3.1.1. Histological Examination

**Figure 1:**
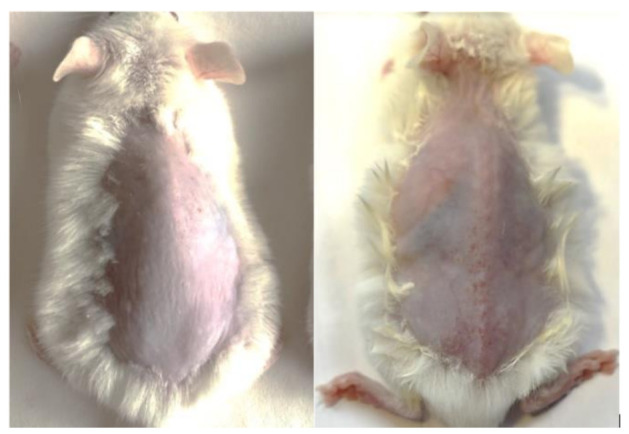
External signs of chronic exposure to UVA in an ICR mouse from the photoaging group (week 12 of the experiment, **left**) compared to the same animal prior to the experiment (**right**).

During the experiment, we were able to trigger chronic UV-induced skin damage in the photoaging group. The obtained result was confirmed by the data from pathomorphological examination, as well as by visual assessment of the animals (Figure 1). During regular external examination, an incremental progression of macroscopic signs of dermal photodamage was observed among the animals from the photoaging group. Particularly, at the last week of the experiment, the animals showed hyperemia of the skin on their dorsa and auricles, ulceration of the auricles and at the tail base area, elastance loss, skin dryness and exfoliation at the dorsal region, wrinkle formation, and eye injuries.

**Figure 2:**
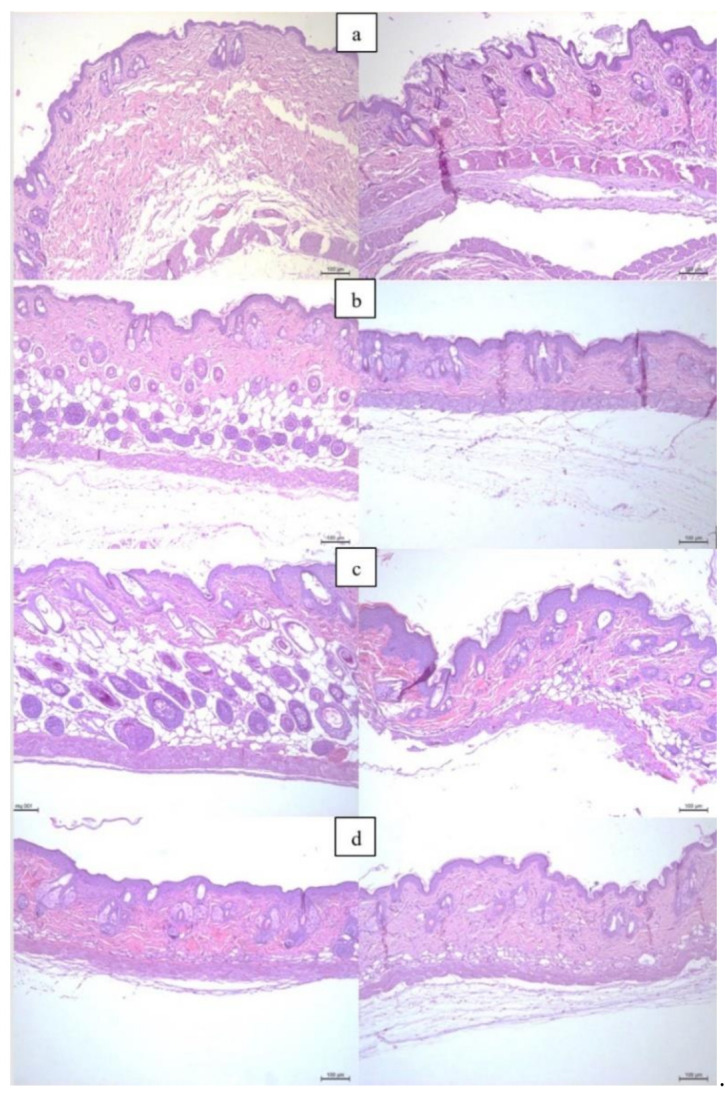
Histological examination of the skin of ICR mice from the photoaging group (**left**) and the control group (**right**): at the beginning of the experiment (without irradiation) (**a**), and after 5 (**b**), 10 (**c**), and 12 (**d**) weeks of the experiment. Hematoxylin and eosin staining, ×100.

Histological examination utilizing hematoxylin and eosin staining revealed incremental signs of photoaging, depending on the cumulative dose of UV exposure: stratum corneum thickening, the appearance of “sunburn cells” (keratinocytes undergoing apoptosis due to UV exposure), edematous and dermal matrix degeneration loci, progressive elastosis, dermal thinning, persistent vasodilation, and inflammatory infiltration (Figure 2). Erosive defects were observed in a number of preparations. It is important to note that the described pathomorphological patterns were uneven, depending on the specific skin reaction to UV exposure, as well as on the experimental conditions. The maximal severity of UV-induced pathomorphological changes was recorded at week 12 of the experiment, which was consistent with visual signs of UV damage. The results obtained are consistent with the data of studies devoted to the use of animal models to initiate the process of photoaging [39].

#### 3.1.2. Optical Diagnostics Results

**Figure 3:**
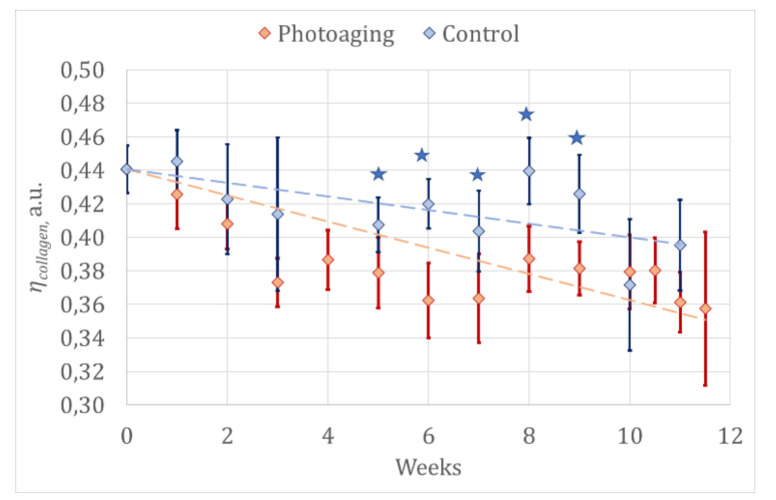
The dynamic of mean values of tissue content indices of collagen *η_collagen_* in animals from the control group and the photoaging group; 95% confidence intervals are specified. Asterisks indicate significant differences between the groups according to the Mann–Whitney test (*p* < 0.05).

The results of laser fluorescence spectroscopy are presented in Table 1 and Figure 3.

It should be noted that calculated values of *η_porphyrins_* were low, and there was no obvious significant difference in the mean values of *η_porphyrins_* between the animal groups. The data obtained indicate the absence of any dynamics; however, they are likely associated with methodological errors—in particular, connected with possible variability in the position of the sensor, its pressure on the tissue, etc.

The results obtained may be related to the fact that chronic long-term exposure to long-wave UVA radiation leads to prevalent atrophy and destruction of collagen fibers in the dermis. At the same time, the inflammatory reaction is weak (in contrast to acute UVB irradiation), which is why *η_porphyrins_* for normal and irradiated skin are low and there is no difference between them. Meanwhile, it was shown that porphyrin autofluorescence can be considered to be a marker of the inflammatory response’s severity. Porphyrins have been previously shown to accumulate in tissues sensitive to ischemia, hypoxia, and inflammation [27]. Their source may be related to the free heme, whose concentration severely increases in tissues due to hemolysis or excessive cell damage induced by internal or external stimuli [40]. Experiments using the LFS conducted by et al. revealed porphyrins’ accumulation in the area of artificially induced skin inflammation in Wistar rats [41]. It is important to note that we previously demonstrated that the autofluorescence of porphyrins reflects the severity of inflammatory infiltration in the skin induced by acute UVB exposure [42].

A similarly designed paper by de Paula Campos et al. presents the results of a comparative analysis of the endogenous fluorescence of hairless HRS/J mice’s skin in a photoaging group (exposed to systematic UV irradiation) and a control group. The fluorescence was excited by a diode laser with a wavelength of 408 nm. The normalized porphyrin fluorescence intensities decreased during the experiment, but the maxima of the recorded porphyrin fluorescence were weakly expressed in contrast to the main peak corresponding to the fluorescence of flavin adenine dinucleotide (FAD), lipids, and other endogenous fluorophores. The authors suggested that the decrease in porphyrin fluorescence during the experiment in nonirradiated skin was due to a decrease in tissue metabolic activity over time, and that the almost unchanged parameters of porphyrin fluorescence in the irradiated zone were due to hyperplasia of the epidermis caused by UV exposure associated with age-related skin changes [17].

Starting from the fifth week, there is a significant difference in *η_collagen_* in the photoaging group compared to the control group, which could be explained by collagen degradation and the development of elastosis under the influence of UV light (Figure 3). The absence of such differences starting from 10th week could be explained by a diminution in the sample size due to the withdrawal of animals for histological examination and anesthetic mortality (by the 10th week, only eight animals in the photoaging group and three animals in the control group remained in the experiment). The 30% of the animals remaining may be not representative enough for correct statistical analysis. Moreover, it should be noted that structures lying beneath the point of the fiber’s contact with tissue (the area under examination) contribute to fluorescent signals, and can cause accidental errors. Therefore, collagen degradation assessment must be carried out throughout the whole experiment, and regression analysis is well suited to such purposes.

To evaluate the rate of collagen degradation, a regression analysis was carried out in the experimental and control groups. The rate of collagen degradation was approximated by the linear function *η_collagen_ = kt + b*, where *t* is the time of the experiment measured in weeks, and *k* and *b* are the coefficients of the linear equation presented in Table 2.

Changes in the fluorescence signals of collagen and elastin have long been considered in the literature to be a marker of chronic UV exposure and photoaging of the skin [19,43]. Furthermore, it is reported that the autofluorescence of NADH and FAD, representing metabolic changes in tissues, can also be used to characterize skin aging [44]. It should be noted that in the studies described, collagen autofluorescence was typically assessed once; the studies included human test subjects and, in addition, there was variability in the methodology (for example, different anatomical zones for analysis) and in the population of the study. There was no histological verification of the optical data. Meanwhile, we were able not only to demonstrate a dynamic change in collagen autofluorescence in a long-term experiment (12 weeks with weekly monitoring) using a standardized animal model, but also, based on mathematical data processing, to propose a quantitative criterion for photoaging—the rate of collagen degradation. In addition, we compared changes in the parameters of collagen fluorescence with the morphological images of the skin at different stages of the experiment (see below).

1. Laser Doppler flowmetry (LDF) and optical tissue oximetry

**Figure A5:**
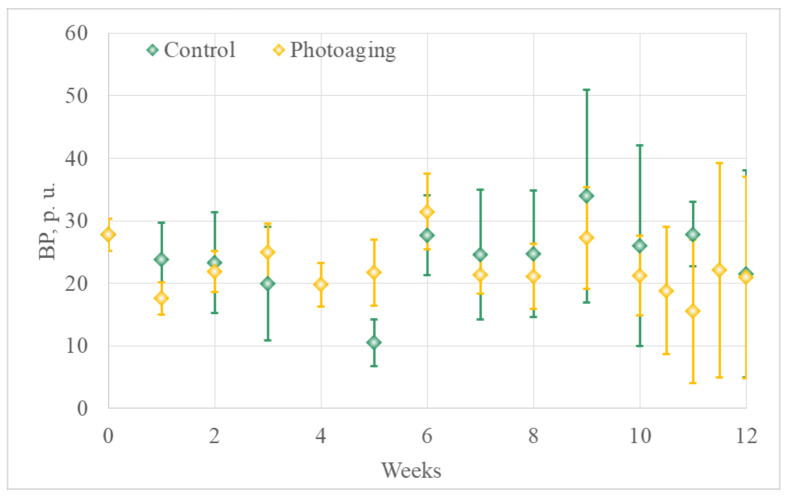
Dynamics of mean values of baseline perfusion (BP) in animal groups measured in perfusion units (p.u.). Standard deviations are indicated.

**Figure A6:**
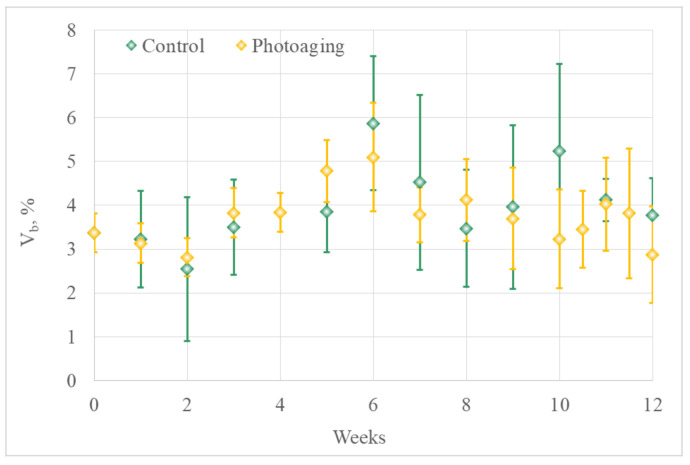
Dynamics of mean values of relative volume of total hemoglobin in a tested tissue’s volume (*V_b_*). Standard deviations are indicated.

No significant and regular differences in average BP and *V_b_* were observed between the groups. During the experiment, a wide scatter in the monitored parameters was observed (for a number of animals, the standard deviations of the registered BP from different areas of the skin of the dorsum exceeded 50%) (Figure A5 and Figure A6).

The penetration depth of the LD signal was about 1 mm, and the measurement result strongly depended on the thickness of the skin [45]. Murine skin is significantly thinner than human skin [46], and subcutaneous structures probably played a significant role in the results of animal tissue measurements. The result could also have been influenced by vessels greater than those defined as belonging to microcirculation [45]. Such a large variability of parameters may be due to the fact that BP has a high spatial and temporal variability, and depends on many factors (sensor tilt, the degree of contact between the sensor and the skin, time of day, food intake, etc.). The technique of BP measurement by LDF is operator-dependent; in the described experiment, perfusion measurement was performed by two trained researchers, which could also contribute to the variability of the studied parameter. Thus, in this experiment, we showed low diagnostic value of microcirculation measurements performed on mice, but this does not mean that the method is inapplicable to humans.

It should be noted that age-related changes in the microcirculation parameters were demonstrated in clinical studies with the participation of volunteers. Thus, in a study involving 50 women aged 20–74 years, using videocapillaroscopy and LDF, it was found that the density of capillary loops in the older group was about 40–70% lower compared to that in younger participants, while the length of the vessels increased by 35–156%. Blood flow levels and the erythema index (a*) also increased with age [47]. When analyzing the scientific literature, we did not manage to find investigations using photoaging animal models, nor longitudinal studies that would allow us to draw a conclusion concerning changes in the microvasculature during long-term observation, as well as to differentiate microcirculatory changes typical of photo- and chronoaging. Most of the published works are focused on the analysis of the short-term effect of various biologically active substances on the microcirculation status of the skin affected by UV exposure [48].

2. The comparison of the results of optical diagnostics and histological examination

**Figure 4:**
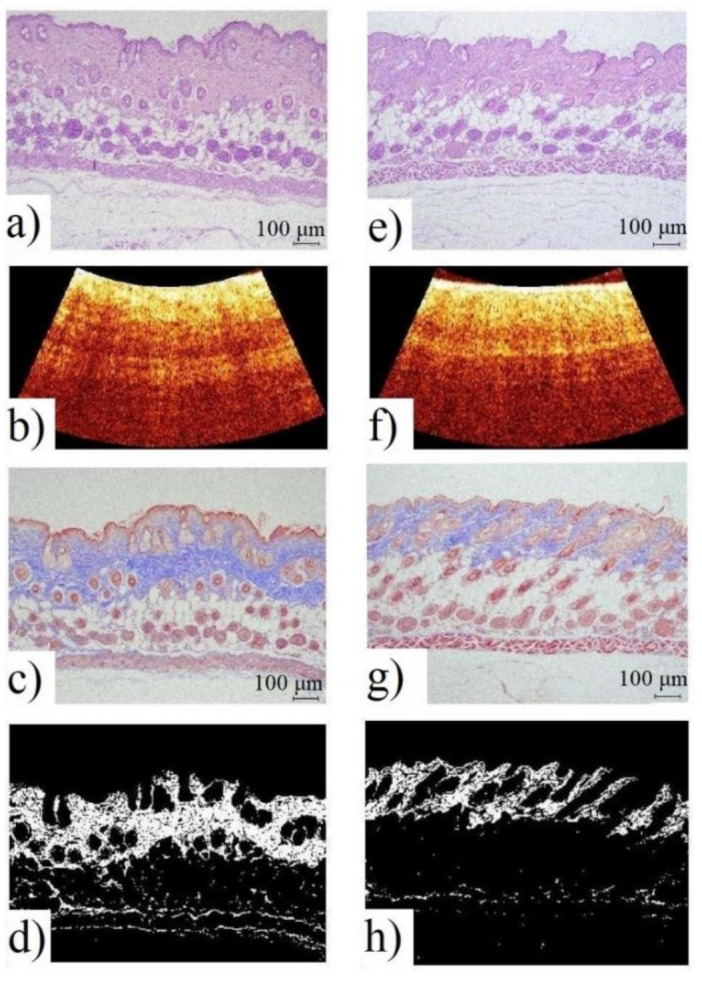
Images of the skin of mice from the “control” (**left**) and “photoaging” (**right**) groups 5 weeks after the start of UV irradiation: histology with hematoxylin/eosin staining (**a**,**e**); OCT (**b**,**f**); histology with Masson’s trichrome staining (**c**,**g**); the result of color threshold filtering of images (**d**,**h**).

During the experiment, OCT images of animal skin were analyzed in parallel with a series of histological preparations. Figure 4 shows OCT images and histological sections with hematoxylin/eosin staining and Masson’s trichrome staining, followed by assay of the percentage of collagen fibers stained in the biological tissue layer.

The OCT study allowed us to compare the obtained images with histological preparations, and to identify the main optical layers: the epidermis, dermis, subcutaneous fat, and muscle layer.

To calculate the area of collagen in the histological section, color threshold filtering was applied to the Masson-colored images. At the fifth week of the experiment, there was a significant decrease of 0.12 ± 0.04 mm^2^ in the area of collagen fibers in the histological section in the “photoaging” group, compared to the control group, with a decrease of 0.22 ± 0.01 mm^2^ (*p* < 0.001). On the remaining days of histological examination (weeks 0, 10, and 12), no significant differences were observed. It is important to note that the obtained data are comparable to the results of the OCT, as well as of the LFS, given above. This confirms the applicability of a set of complementary optical methods for the highly specific diagnostics of skin changes caused by chronic UV exposure.

The obtained results correlate with the previously published data and supplement them. A number of studies show that OCT is capable of measuring characteristic skin changes—such as the thickness and elasticity of the epidermis—that occur during age-related skin involution [49,50,51]. For example, OCT has been shown to detect changes in the thickness of the epidermis in the forehead, chest, forearm, scapular, and gluteal regions that correlated with the age of patients [49]. It is known that OCT is capable of quantifying the increase in epidermal thickness, dermal edema, and the degree of vasodilation [50].

A limited number of available studies consider the correlation between the parameters obtained by various optical methods and the histological skin changes associated with extended UV exposure. In a similarly designed study, Papazoglou et al. compared LFS data, the morphological study, and the expression of matrix metalloproteinase 13 (MMP-13) to assess changes caused by extended exposure to UVB radiation on the skin of hairless mice. The authors found that to assess the thickness of the epidermis, laser fluorescence spectroscopy can be used, which correlates with tryptophan expression and cell proliferation, and may indicate the presence of “sunburn cells” (apoptotic keratinocytes) in the epidermis [24]. Wu et al., in their study of a murine model of photo- and chronoaging using single-scattering OCT, obtained quantitative data and compared them with the histological pattern; significant differences between damping factors for both types of aging were found in the core of the epidermis [51].

### 3.2. Colorimetric Assay of Erythema from an Image Using a Digital RGB Camera

**Figure 5:**
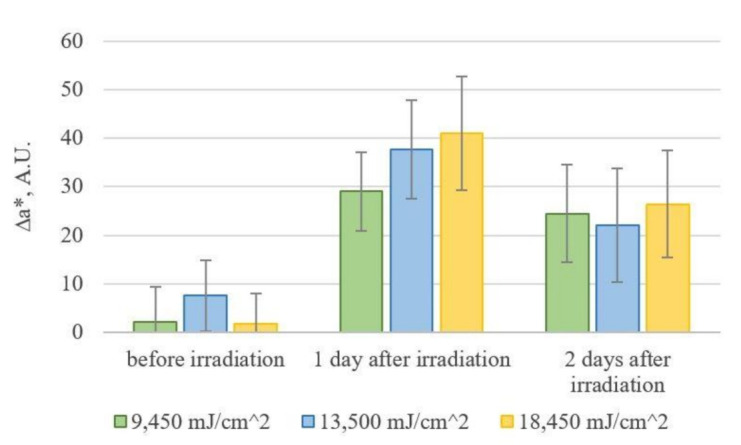
Dynamics of mean values of ∆a* changes by group.

**Figure 6:**
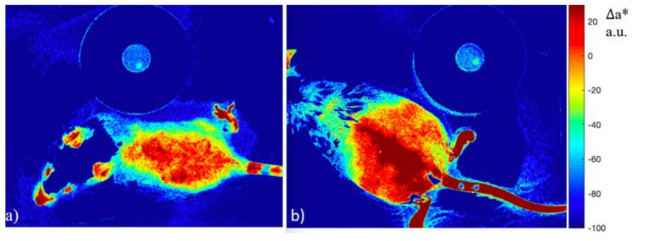
The results of ∆a* calculation for a mouse from the group that received a dose of 9450 mJ/cm^2^ prior to irradiation (**a**), and on the day after irradiation (**b**).

During image processing, the nonirradiated (right) part of the murine dorsum was taken as the intact region. Figure 5 shows the ∆a* indices obtained from photos of groups of mice that received different doses of UVB radiation. The visualization of the ∆a* index for the mouse M2D1 in the group that received a dose of 9450 mJ/cm^2^ is shown in Figure 6.

After irradiation, the ∆a* index increases, and never returns to the initial level. The statistical analysis with pairwise comparisons according to Tukey’s criterion showed the presence of statistically significant differences between ∆a* index values within each of the groups on the days prior to and after exposure. The differences at *p*-values <0.05 were considered significant. At the same time, no significant differences were found between 1 and 2 days after exposure, nor were any differences found between the groups that received different doses. Thus, the considered method for evaluation of the relative erythema index ∆a* is limited in sensitivity to differentiate groups that received UVB radiation doses of 9450 mJ/cm^2^, 13,500 mJ/cm^2^, and 18,450 mJ/cm^2^. However, this method allows us to establish the existence of a skin reaction to irradiation that is not visible to the naked eye, and to visualize it in the image.

Methods utilizing a digital camera show a high correlation with the results of other UV-induced erythema assays, such as diffuse scattering spectroscopy. However, slight differences due to various radiation doses are not always detected by either method [52]. Difficulties in dose differentiation also arise with such methods as fluorescence spectroscopy or optoacoustic mesoscopy [24,53]. However, each of these methods is more informative and objective for the assessment of skin reactions in the formation of UV-induced erythema than the generally accepted visual assessment.

### 3.3. Limitations of Study

It should be noted that the present study on the optical assessment of photoaging has a number of limitations, including the sample size (during the latter stages of observation period), the duration of the observation period, and the lack of highly specific immunohistochemical and immunofluorescence techniques. Thus, more data can be collected by increasing the study population and the duration of exposure, by improving the survival of animals at each stage, and by using immunohistochemical staining of specimens for the dynamic assessment of specific photoaging markers (e.g., matrix metalloproteinases, certain types of collagen) and comparing them with optical data. In addition, the results of the acute UV damage assessment could be improved by involving more animals, as well as by using a wider range of UVB doses.

## 4. Conclusions

In this paper, we presented and analyzed data from a weekly comprehensive optical assessment of animal skin with the use of LFS, LDF/OTO, and OCT in parallel, identified significant optical markers of photoaging (collagen fluorescence), compared their dynamic changes in compliance with the histological pattern, and developed a criterion for the differential diagnosis of photoaging and chronoaging.

The main result of this work is the development of an equation for comparative evaluation of the collagen degradation rate, based on the LFS assay and pathomorphological data. In addition, the OCT results correlated well with the histological data, which makes the technique promising. The potential applicability of the RGB camera in the assessment of acute UV-induced erythema was also demonstrated; it is superior in accuracy to visual inspection, and allows the analysis of the image together with the evaluation of the erythema index.

The obtained data are of practical importance, and can be further used to determine the therapeutic dose of UV exposure, to formulate clinical algorithms for diagnosing early signs of photoaging or pathological photosensitivity, and to identify groups of patients with an increased risk of malignant skin tumors. In addition, the results can be applied in the pharmaceutical industry to test the antiaging effects and SPF factor of cosmetics.

## Figures and Tables

**Table 1:** Comparison of tissue content indices of porphyrins *η_porphyrins_* in skin tissue between animals from the control group and the photoaging group. Means and standard deviations are indicated. *p*-values were calculated with the Mann–Whitney test.

Week	Control Group	Photoaging Group	*p*-Value *
1	0.05 ± 0.01	0.06 ± 0.02	0.328
2	0.06 ± 0.01	0.06 ± 0.01	0.401
3	0.06 ± 0.01	0.07 ± 0.02	0.709
5	0.04 ± 0.01	0.04 ± 0.01	0.123
6	0.05 ± 0.01	0.04 ± 0.01	0.180
7	0.04 ± 0.01	0.05 ± 0.01	0.037 *
8	0.04 ± 0.01	0.05 ± 0.01	0.256
9	0.04 ± 0.01	0.03 ± 0.01	0.049 *
10	0.04 ± 0.01	0.05 ± 0.02	0.776
11	0.04 ± 0.01	0.04 ± 0.01	0.548
12	0.05 ± 0.01	0.04 ± 0.01	0.400

* *p* < 0.05.

**Table 2:** Linear regression equation coefficients *η_collagen_(t)*.

Group		Value	Standard Error	*p*-Value	95% Confidence Interval	
					Bottom Border	Upper Border
Control	*b*	0.431	0.008	<0.001 *	0.415	0.446
	*k*	−0.003	0.001	0.0021 *	−0.0053	−0.0005
Photoaging	*b*	0.420	0.005	<0.001 *	0.411	0.430
	*k*	−0.006	0.001	<0.001 *	−0.0075	−0.0041

* *p* < 0.05.

## Data Availability

Not applicable.
